# Cartilage’s Contribution in Otology: A Comprehensive Review of Its Role in Ear Surgery

**DOI:** 10.7759/cureus.49800

**Published:** 2023-12-01

**Authors:** Jasleen Kakkad, Prasad Deshmukh, Sagar Gaurkar

**Affiliations:** 1 Otolaryngology, Head and Neck Surgery, Jawaharlal Nehru Medical College, Datta Meghe Institute of Higher Education and Research, Wardha, IND

**Keywords:** regenerative medicine, 3d printing technology, biodegradable scaffolds, tissue engineering, cartilage, otologic surgery

## Abstract

This comprehensive review thoroughly examines the pivotal role of cartilage in otologic surgery, elucidating its multifaceted contributions to both cosmetic and functional outcomes. From reconstructing the external ear to reinforcing the tympanic membrane and restoring the ossicular chain, cartilage emerges as a versatile and resilient biological material with unique properties that make it an invaluable resource for otologic surgeons. The review explores the nuances of cartilage's applications in various surgical contexts, emphasizing its significance in promoting tissue regeneration and healing. The text delves into advancements in tissue engineering, biodegradable scaffolds, and 3D printing technology, pointing toward a future where more precise and personalized interventions may redefine the landscape of otologic surgery. The convergence of these innovations holds the promise of elevating the standard of care, minimizing complications, and improving the quality of life for patients undergoing cartilage-based otologic procedures. This synthesis of current knowledge and future possibilities provides a valuable resource for otologists, surgeons, and researchers in the dynamic field of otology.

## Introduction and background

Otology, a specialized branch of medicine focused on studying and treating disorders related to the ear, plays a crucial role in preserving auditory function and maintaining overall ear health. The field encompasses many conditions affecting the ear's external and internal structures, demanding a nuanced understanding of anatomy, physiology, and surgical techniques. As the pursuit of effective interventions for various ear-related ailments continues, there has been a growing appreciation for cartilage's role in otologic surgery [1].

Cartilage, a flexible and resilient connective tissue, is a versatile biological material with unique properties suitable for many applications in ear surgery. The importance of cartilage in otologic procedures stems from its ability to provide structural support, promote tissue regeneration, and enhance overall surgical outcomes. Cartilage has proven invaluable for otologic surgeons, whether utilized in reconstructive procedures for the external ear, tympanic membrane, or ossicular chain [2].

This comprehensive review aims to delve into the multifaceted contributions of cartilage in otology, offering a detailed examination of its role in various surgical contexts. By synthesizing current knowledge and highlighting recent advancements, this review aims to provide otologists, surgeons, and researchers with a comprehensive resource that outlines the anatomical and physiological aspects of cartilage within the ear and explores its applications in diverse surgical scenarios. Through an in-depth analysis of surgical techniques, outcomes, complications, and future directions, this review seeks to contribute to the existing body of literature, fostering a deeper understanding of the intricate interplay between cartilage and otologic surgery. By elucidating the pivotal role that cartilage plays in addressing structural deficiencies, promoting healing, and enhancing functional restoration, this review aims to serve as a valuable reference for both novice and seasoned practitioners in otology.

## Review

Types of cartilage in the ear

Auricular Cartilage

The auricle, or external ear, owes much of its structural integrity to auricular cartilage. Comprising a flexible yet resilient framework, auricular cartilage provides the unique and intricate contours of the pinna. This anatomical prominence is not merely aesthetic; it plays a crucial role in sound localization and the overall functionality of the auditory system. Auricular cartilage is frequently harnessed in surgical procedures aimed at reconstructing the external ear, with notable applications in correcting congenital malformations such as microtia and creating frameworks for prosthetic ears. Understanding the properties and nuances of auricular cartilage is paramount for ear reconstruction surgeons [3].

Tympanic Membrane Cartilage

The tympanic membrane, or eardrum, separates the external ear from the middle ear and is pivotal for transmitting sound waves. In certain otologic surgeries, cartilage can reinforce or repair the tympanic membrane. Grafts of cartilage, often harvested from the tragus or other auricular sources, can be meticulously shaped to augment the structural integrity of the tympanic membrane. This application becomes particularly relevant in cases of chronic perforations, where traditional tympanoplasty techniques may benefit from the resilient and supportive characteristics of cartilage [4].

Cartilage in the Ossicular Chain

The ossicular chain, consisting of the malleus, incus, and stapes, is the crucial intermediary between the tympanic membrane and the inner ear. Cartilage finds applications in reconstructive procedures involving the ossicular chain, addressing disruptions caused by trauma, infection, or congenital anomalies. Ossicular chain reconstruction often necessitates cartilage grafts to replace or augment damaged or missing ossicles. The structural strength and biocompatibility of cartilage make it an ideal material for such intricate and functionally critical interventions. Understanding the biomechanical considerations and surgical techniques associated with ossicular chain reconstruction is fundamental for successful outcomes in otologic surgery [5]. Exploring the distinct roles of auricular cartilage, tympanic membrane cartilage, and cartilage in the ossicular chain provides a foundation for comprehending the diverse applications of cartilage in ear surgery. Each type of cartilage presents unique anatomical, biomechanical, and surgical considerations, underscoring the need for a nuanced understanding of these variations to optimize surgical outcomes and enhance patient well-being [6].

Role of cartilage in otologic surgery

Auricular Reconstruction

Microtia repair: Microtia, a congenital deformity characterized by underdeveloped external ears, presents a complex challenge that requires meticulous surgical intervention. Cartilage grafts, typically sourced from the patient's rib or contralateral ear, play a crucial role in microtia repair. These grafts are intricately shaped and sculpted to recreate the auricle's intricate contours, ensuring a natural appearance and a stable and resilient framework. The use of cartilage in microtia repair contributes significantly to improved aesthetic outcomes, offering patients a harmonious and balanced ear structure. This approach addresses microtia's functional and cosmetic aspects, ultimately enhancing patient satisfaction [7].

Framework for ear prosthesis: In scenarios where surgical reconstruction may not be feasible or preferred, cartilage emerges as an indispensable material for fabricating ear prostheses. Whether these prostheses are intended as temporary solutions or permanent replacements, cartilage serves as the foundational scaffolding to replicate the natural shape and contours of the ear. The versatility of cartilage allows for the customization of prostheses, ensuring that they not only fulfill functional requirements but also achieve a cosmetically pleasing result. By providing a framework for ear prostheses, cartilage offers patients a viable and personalized alternative, addressing ear restoration's physical and aesthetic dimensions [8].

Tympanoplasty

Grafting techniques: Cartilage grafts, harvested from reliable sources such as the tragus or conchal bowl, emerge as a dependable and structurally robust option for tympanic membrane repair. Cartilage is pivotal in facilitating healing, whether utilized as an underlay or overlay graft. Its mechanical strength and resistance to resorption position cartilage are ideal for tympanoplasty, especially in scenarios involving large perforations or when substantial support is essential. The versatility of cartilage grafting techniques allows for tailored solutions, contributing to the success of tympanic membrane repair and addressing challenges associated with compromised membrane integrity [9].

Reinforcement of the tympanic membrane: In situations where the native tympanic membrane exhibits weakness or thinning, cartilage reinforcement is a valuable augmentation to conventional grafting techniques. The addition of structural support through cartilage significantly reduces the risk of graft failure and enhances the long-term stability of the repair. This is particularly pertinent in cases of chronic otitis media, where recurrent infections may compromise the natural integrity of the tympanic membrane. The reinforcement provided by cartilage fortifies the membrane against potential stressors. It contributes to improved outcomes in managing chronic middle ear conditions, offering a robust solution to challenges associated with tissue fragility [10].

Ossicular Reconstruction

Use of cartilage in ossicular chain reconstruction: Cartilage emerges as a valuable material in the intricate process of ossicular chain reconstruction, often employed to craft prostheses. Unlike traditional materials, cartilage offers a lightweight and biocompatible alternative, contributing to restoring middle ear function. Whether utilized as total or partial ossicular replacement prostheses, cartilage grafts establish a stable connection between the remaining ossicles, thereby facilitating the transmission of sound waves through the middle ear. This application of cartilage in ossicular chain reconstruction represents a significant advancement in restoring auditory function with materials that harmonize seamlessly with the natural environment of the middle ear [11].

Functional outcomes: Incorporating cartilage into ossicular reconstruction procedures has yielded promising functional outcomes, as evidenced by studies reporting improved hearing thresholds and heightened patient satisfaction. Cartilage's ability to integrate seamlessly into the middle ear environment is complemented by its resistance to resorption, ensuring the long-term success of ossicular reconstruction. The favorable functional outcomes observed in patients undergoing these procedures underscore the efficacy of cartilage in promoting sound transmission and addressing auditory deficits. The use of cartilage contributes to the structural stability of the reconstructed ossicular chain and enhances the overall quality of life for individuals experiencing hearing impairments, marking a significant stride in otologic surgery [12].

Surgical techniques utilizing cartilage

Cartilage Harvesting

Autologous cartilage: The utilization of autologous cartilage represents a cornerstone in otologic surgery, providing a reliable and biocompatible source for grafting. Frequently harvested from the concha of the ear or the rib, autologous grafts offer inherent compatibility with the patient's tissue. This significantly reduces the risk of immunological reactions and enhances the overall success of graft integration. Notable examples of autologous cartilage types employed in otologic procedures include auricular cartilage, valued for its flexibility and curvature-matching properties, and costal cartilage, known for its robustness and suitability for larger grafts. Autologous cartilage is a testament to the field's commitment to personalized and patient-specific solutions, ensuring optimal outcomes in various reconstructive endeavors [11].

Allograft cartilage: Allograft cartilage, sourced from cadaveric donors, provides a viable alternative for patients facing challenges related to insufficient autologous cartilage or those seeking to circumvent additional donor site morbidity. While allografts offer potential advantages, such as availability and reduced surgical invasiveness, careful consideration is paramount. Factors such as immunogenicity and the risk of disease transmission necessitate thorough evaluation before opting for allograft options. Despite these considerations, allograft cartilage is an invaluable resource, broadening the scope of possibilities in otologic surgery and addressing the diverse needs of patients with varying anatomical and medical profiles. The judicious weighing of risks and benefits ensures that allografts contribute to the field's commitment to providing effective and safe solutions for patients requiring cartilage-based interventions [13].

Minimally Invasive Approaches

Endoscopic cartilage harvesting: Endoscopic cartilage harvesting represents a paradigm shift in otologic surgery, leveraging advanced technology to achieve precise visualization and targeted cartilage retrieval through smaller incisions. This innovative approach minimizes disruption to surrounding tissues, providing a more conservative alternative to traditional open procedures. The use of endoscopic technology not only facilitates meticulous cartilage harvesting from sources such as the concha or rib but also contributes to a reduction in postoperative discomfort for patients. By optimizing visualization and minimizing tissue trauma, endoscopic cartilage harvesting is a testament to the evolving landscape of minimally invasive techniques in otologic surgery, enhancing both the surgical experience and patient outcomes [14].

Arthroscopic techniques: Borrowing principles from arthroscopy, otologic surgeons are increasingly adopting minimally invasive arthroscopic techniques for cartilage retrieval. This approach, inspired by joint surgery practices, offers distinct advantages over traditional open methods. Arthroscopic procedures provide enhanced visualization of the cartilage source, allowing surgeons to navigate with precision and efficiency. The reduced invasiveness associated with arthroscopic techniques translates to minimized tissue disruption, potentially leading to faster recovery times and decreased postoperative morbidity. As otology embraces the benefits of arthroscopic principles in cartilage harvesting, the field continues to evolve toward more refined and patient-friendly approaches, setting the stage for continued advancements in minimally invasive otologic surgery [15].

Cartilage Shaping and Carving

Framework design: The meticulous design of the cartilage framework stands as a pivotal aspect in otologic surgery, requiring surgeons to carefully tailor each graft to meet the precise anatomical requirements of the reconstructive procedure at hand. Whether the goal is replicating the intricate contours of the external ear or reconstructing components of the ossicular chain, the surgeon's skill in framework design directly influences the success of the surgical outcome. Achieving anatomical fidelity contributes to cosmetic harmony and ensures the newly reconstructed ear's functionality and structural integrity. This intricate process underscores the importance of a surgeon's artistry and anatomical understanding in crafting a cartilage framework that seamlessly integrates with the patient's unique anatomy [16].

Graft tailoring: Precision in graft tailoring represents a critical phase in otologic surgery, where the surgeon's attention to detail directly impacts both cosmetic aesthetics and functional efficacy. Various techniques, including scoring, notching, and carving, refine the cartilage graft's shape, size, and flexibility. Scoring allows controlled shaping, notching aids in achieving specific contours, and carving refines the graft's overall structure. These tailored modifications ensure that the cartilage graft aligns seamlessly with the intricate anatomy of the recipient site. By customizing the graft to the specific needs of the patient and the surgical goal, surgeons enhance the likelihood of achieving optimal outcomes, where both form and function harmonize to restore natural appearance and maintain or improve auditory function. The artistry involved in graft tailoring epitomizes the precision and finesse required in otologic surgery [17].

Advances in Surgical Instrumentation

Microsurgical instruments: Using high-precision instruments represents a cornerstone in refining cartilage shaping techniques. These specialized tools empower otologic surgeons to work with unparalleled accuracy, facilitating intricate incisions and adjustments during cartilage shaping. The microscopic scale of these instruments allows for meticulous control, enabling surgeons to achieve precise contours and structures. By harnessing the capabilities of microsurgical instruments, surgeons elevate the level of precision in cartilage shaping, ensuring that the graft aligns seamlessly with the unique anatomy of the recipient site. This emphasis on accuracy underscores the importance of technological advancements in instrument design, contributing to the overall success of otologic procedures and enhancing the surgeon's ability to achieve optimal outcomes [18].

Computer-Aided Design and 3D printing: Integrating Computer-Aided Design (CAD) technologies and 3D printing has ushered in a new era in cartilage shaping, offering a paradigm shift in otologic surgery. Surgeons can now leverage CAD software to plan and design patient-specific grafts tailored to individual anatomy meticulously. This personalized approach allows unparalleled precision in shaping cartilage, ensuring an exact fit and optimal functionality. The subsequent use of 3D printing technology transforms these digital designs into tangible, 3D structures, providing surgeons with physical models for preoperative planning and intraoperative guidance. This innovative combination of CAD and 3D printing enhances surgical precision and improves overall outcomes, exemplifying the transformative impact of technology in advancing the frontiers of otologic surgery [19].

Complications and limitations

Graft Resorption: Factors Influencing Resorption

Graft thickness: The thickness of cartilage grafts emerges as a critical consideration in otologic surgery, as it directly influences the potential for graft resorption. Thinner cartilage grafts may be more prone to resorption due to reduced vascularization and nutrient diffusion while offering flexibility and ease of manipulation advantages. Surgeons are tasked with striking a delicate balance between achieving the desired graft thickness and maintaining structural integrity. This delicate equilibrium ensures that the graft provides adequate support for the intended reconstructive purpose while minimizing the risk of complications associated with resorption. The consideration of graft thickness underscores the surgeon's nuanced decision-making, emphasizing the need for a tailored approach to each patient's unique anatomical and functional requirements [20].

Graft site: The site of graft placement plays a pivotal role in determining the resorption rate of cartilage grafts. Variations in vascular supply and tissue characteristics at different recipient sites can significantly impact the success of graft integration. Surgeons must thoroughly understand the recipient site's vascularisation and tissue properties to minimize the risk of resorption. By carefully selecting an appropriate graft site, surgeons can optimize the chances of successful graft survival and long-term stability. This consideration emphasizes the importance of individualized surgical planning, where the choice of graft site is tailored to each patient's specific anatomy and the requirements of the reconstructive procedure. The nuanced evaluation of graft sites contributes to cartilage-based interventions' overall success and durability in otologic surgery [21].

Graft Resorption: Mitigation Strategies

Perichondrial preservation: The preservation of the perichondrium emerges as a crucial technique in cartilage graft harvesting, significantly impacting graft survival. The perichondrium, a thin layer of connective tissue surrounding cartilage, contains vital blood vessels that provide vascular support for graft nourishment. Techniques focused on preserving the perichondrium during harvesting contribute to the maintenance of this vascular network. By safeguarding the perichondrium, surgeons enhance the chances of graft survival, as the preserved vascular support facilitates nutrient diffusion and fosters the graft's integration with the recipient site. This nuanced approach to perichondrial preservation exemplifies the importance of meticulous surgical techniques in optimizing the success of cartilage grafts in otologic surgery [22].

Suturing techniques: How cartilage grafts are sutured into place plays a pivotal role in preventing graft resorption and ensuring long-term stability. Proper suturing methods are essential, and surgeons must exercise precision to avoid excessive tension on the graft. Excessive tension can compromise blood supply, leading to resorption and jeopardizing the overall success of the graft. Additionally, adequate graft stabilization through proper suturing techniques is paramount for preventing displacement and promoting optimal healing. The choice of suture materials, the placement of sutures, and the tension applied during suturing collectively contribute to the success of the graft integration process. Through meticulous attention to suturing details, surgeons can mitigate graft complications risk and enhance cartilage-based interventions' overall outcomes in otologic surgery [23].

Infection Risks: Contamination During Harvesting

Aseptic techniques: Maintaining strict adherence to aseptic techniques during cartilage harvesting is paramount in otologic surgery. Aseptic protocols are crucial for minimizing the risk of contamination, ensuring that the harvested cartilage remains free from microbial pathogens. Contamination during the harvesting process can lead to postoperative infections and compromise the overall success of the graft integration. Surgeons and their teams must rigorously follow sterile procedures, including sterile instruments and a sterile environment, to safeguard the integrity of the cartilage graft. The emphasis on aseptic techniques underscores the commitment to preventing infections and promoting optimal healing in otologic surgical procedures [24].

Donor site infection: In autologous grafts, where the cartilage is harvested from the patient's body, the risk of infection at the donor site is a critical consideration. Whether the graft is obtained from the rib or the concha, infection at the donor site can have implications for both the donor and recipient sites. Donor site infections not only threaten the patient's overall health but can also compromise the quality and viability of the harvested cartilage. Surgeons must monitor and prevent infections at the donor site, implementing appropriate preoperative and postoperative care protocols. Mitigating the risk of donor site infections is essential to ensure the successful integration of autologous cartilage grafts and to safeguard the overall well-being of the patient undergoing otologic surgery [25].

*Infection Risks:* *Postoperative Infections*

Surgical site infections: Vigilant postoperative monitoring is paramount in preventing and managing surgical site infections following otologic surgery involving cartilage grafts. Careful observation and timely detection of any signs of infection at the surgical site are crucial for preserving graft viability and optimizing patient outcomes. Surgical site infections can pose a significant threat to the success of the graft integration, leading to complications such as graft resorption or structural compromise. Surgeons and healthcare teams must implement rigorous postoperative protocols, including regular assessments and appropriate interventions, to mitigate the risk of surgical site infections and ensure the effective healing of cartilage grafts [26].

Systemic infections: Patients with compromised immune systems represent a distinct population at an elevated risk of systemic infections following otologic surgery. Preoperative screening becomes imperative in identifying and addressing potential systemic infections that could adversely impact the success of cartilage graft procedures. Systemic infections can compromise the healing process and increase the susceptibility to postoperative complications. The emphasis on preoperative screening underscores the proactive approach needed in managing patients with compromised immune function, ensuring that appropriate measures are taken to minimize the risk of systemic infections and optimize the conditions for successful cartilage graft integration. This comprehensive approach to infection prevention is integral to the overall success and safety of otologic surgeries involving cartilage grafts [27].

Patient-specific factors affecting cartilage use

Previous Surgical History

Scar tissue: Patients with a history of multiple surgeries may present increased scar tissue at the surgical site, impacting the vascularity and success of cartilage graft integration. Excessive scar tissue may alter the local tissue environment, affecting the blood supply and nutrient diffusion necessary for graft survival. Surgeons must carefully evaluate the presence of scar tissue during preoperative assessments and consider potential challenges it may pose to successful graft integration. Strategies to address and navigate through scar tissue become essential to optimize the outcomes of cartilage graft procedures in patients with a history of multiple surgeries [28].

Prior infections: Individuals with a history of recurrent infections in the ear may experience compromised tissue quality, introducing additional challenges in cartilage graft procedures. Prior infections can alter the local environment, potentially leading to tissue damage or changes that impact the success of graft integration. Surgeons must consider the patient's medical history, specifically assessing the impact of prior infections on the ear's structural and functional aspects. Managing the consequences of previous infections becomes integral to minimizing the risk of complications and ensuring the success of cartilage graft interventions [29].

Medical Conditions

Connective tissue disorders: Patients with connective tissue disorders pose a unique challenge in cartilage graft integration due to altered tissue characteristics. Conditions such as Ehlers-Danlos syndrome or Marfan syndrome may affect connective tissues' elasticity and strength, potentially influencing graft procedure success. Surgeons must carefully consider the specific challenges associated with connective tissue disorders, adapting surgical techniques and approaches to accommodate the unique characteristics of the patient's tissues [30].

Immune status: Immunocompromised patients, whether due to underlying medical conditions or medical treatments, may experience impaired healing and an elevated risk of infections. The decision to use cartilage grafts in immunocompromised individuals requires careful consideration of the potential impact on healing and the heightened susceptibility to complications. Surgeons must collaborate closely with immunologists and other specialists to assess the patient's immune status and implement tailored strategies to mitigate risks and optimize outcomes in cartilage graft procedures [31].

Innovations and future directions: tissue engineering in cartilage regeneration

Cellular Therapies

Chondrocyte implantation: The innovative approach of chondrocyte implantation represents a promising avenue in regenerative interventions for cartilage defects. This technique involves the introduction of cultured chondrocytes, cartilage cells, into areas of cartilage damage, stimulating the synthesis of native cartilaginous tissue. By implanting these cultured cells directly into the affected site, surgeons aim to enhance the regenerative capacity of the damaged cartilage. Chondrocyte implantation holds great potential for addressing cartilage defects in various anatomical locations, offering a targeted and biologically driven solution that promotes the restoration of functional and resilient cartilaginous tissue. This regenerative strategy represents a significant advancement in the field, showcasing the potential for precise interventions in cartilage repair [32].

Mesenchymal stem cells: Mesenchymal stem cells (MSCs) have emerged as powerful agents in promoting cartilage regeneration. These multipotent cells can differentiate into chondrocytes, the specialized cells responsible for cartilage formation. When introduced into the damaged cartilage area, MSCs contribute to the regeneration process by differentiating into chondrocytes and facilitating the synthesis of new cartilaginous tissue. MSCs exhibit immunomodulatory properties and can modulate the local microenvironment, fostering an environment conducive to tissue repair. Applying MSCs in cartilage regeneration represents a dynamic and versatile strategy, offering a biologically driven approach to address cartilage damage and degeneration. This evolving field holds promise for developing advanced therapies that harness the regenerative potential of stem cells to improve outcomes in cartilage repair and reconstruction [33].

Biomimetic Scaffolds

Use of natural polymers: Integrating natural polymers, including collagen and hyaluronic acid, in scaffolds represents a sophisticated approach to enhance biocompatibility and create a biomimetic environment conducive to cartilage regeneration. Collagen, a significant component of the extracellular matrix, and hyaluronic acid, known for its lubricating and hydrating properties, offer structural support and mimic the natural composition of cartilage. By incorporating these natural polymers into scaffolds, researchers and surgeons aim to create environments that closely resemble the native tissue, fostering cell adhesion, proliferation, and differentiation. Using natural polymers in scaffold design signifies a biologically inspired strategy for cartilage regeneration, promising improved clinical outcomes in cartilage repair [34].

Synthetic polymers: Tailoring synthetic polymers with specific mechanical and degradation properties allows for the creation of customized scaffolds designed to mimic the native cartilage matrix. Synthetic polymers, such as poly(lactic-co-glycolic acid) (PLGA) and polyethylene glycol (PEG), offer versatility in scaffold design, enabling the manipulation of parameters like stiffness, porosity, and degradation rate. The ability to fine-tune these properties facilitates the development of scaffolds tailored to the unique requirements of cartilage regeneration. Synthetic polymers provide a platform for engineering precise and controlled environments that support cell growth and tissue formation. This approach represents a synthetic yet highly customizable cartilage regeneration strategy, contributing to biomaterials' evolution in regenerative medicine [35].

Biodegradable Scaffolds: Polymeric Scaffolds

Polyglycolic acid and polylactic acid: Biodegradable polymers, including polyglycolic acid (PGA) and polylactic acid (PLA), play a pivotal role in scaffold design for cartilage regeneration. These polymers provide mechanical support during the crucial initial stages of healing before gradually breaking down over time. PGA and PLA are known for their biocompatibility and ability to be resorbed by the body, making them ideal candidates for creating scaffolds that facilitate the regrowth of cartilage tissue. As these polymers degrade, they allow for the gradual integration of newly formed tissue, providing a temporary structural framework that aids in regeneration. Using PGA and PLA exemplifies the strategic application of biodegradable polymers in scaffold design to support and guide cartilage healing [36].

Composite scaffolds: Composite scaffolds, formed by combining different biodegradable materials, represent an advanced approach in scaffold design for cartilage regeneration. By blending biodegradable polymers with natural fibers or other materials, researchers can create scaffolds with improved mechanical properties and controlled degradation rates. The synergistic effect of combining materials allows for optimizing scaffold characteristics, such as strength, flexibility, and degradation kinetics. This tailored approach addresses the complex requirements for successful cartilage regeneration, providing a supportive environment for cells to proliferate and differentiate. Composite scaffolds showcase the versatility of biomaterial engineering, offering a multifaceted solution that aims to enhance the overall effectiveness of scaffolds in promoting cartilage repair [37].

Controlled Release Systems

Growth factor delivery: The integration of growth factors into biodegradable scaffolds represents a sophisticated strategy in cartilage regeneration. These growth factors, such as transforming growth factor-beta (TGF-β) or platelet-derived growth factor (PDGF), are crucial for signaling and regulating cellular processes involved in tissue repair. Incorporating growth factors into scaffolds allows for controlled release, creating a microenvironment that promotes cell proliferation and differentiation during the regenerative process. This targeted and sustained delivery of growth factors enhances the biological cues available to cells within the scaffold, encouraging them to contribute to forming functional cartilage tissue. Growth factor delivery within biodegradable scaffolds exemplifies a bioactive approach that harnesses the body's natural healing mechanisms for optimal cartilage regeneration [38].

Drug-eluting scaffolds: Scaffolds that release antimicrobial agents or anti-inflammatory drugs represent an innovative strategy for enhancing overall graft success, particularly in minimizing infection risks. Drug-eluting scaffolds provide a localized and controlled release of therapeutic agents directly to the implant site, reducing the chances of infections that could compromise the graft. Incorporating antimicrobial or anti-inflammatory drugs into the scaffold material creates a microenvironment that discourages infection and inflammation, thereby improving the overall success of the graft. This approach showcases the dual functionality of drug-eluting scaffolds, offering structural support for tissue regeneration and a protective mechanism against potential complications, reinforcing the scaffold's role in facilitating successful cartilage repair [39].

Advances in 3D Printing Technology: Patient-Specific Grafts

Imaging-based planning: Integrating imaging data into cartilage reconstruction processes represents a revolutionary approach, allowing for the creation of 3D-printed grafts precisely tailored to the patient's unique anatomy. Advanced imaging techniques, such as computed tomography (CT) scans or magnetic resonance imaging (MRI), provide detailed information about the patient's cartilage structure and defects. Surgeons can use this imaging data to generate accurate 3D models of the affected area, which can be translated into custom-designed grafts through 3D printing. This imaging-based planning approach significantly improves the precision of graft design, ensuring a seamless fit and alignment with the patient's anatomy. Using imaging data in conjunction with 3D printing exemplifies a paradigm shift toward personalized and accurate solutions in cartilage reconstruction, ultimately enhancing surgical outcomes [40].

Rapid prototyping: 3D printing technology enables the rapid prototyping of custom implants, introducing a more efficient and personalized approach to cartilage reconstruction. This innovative method lets surgeons quickly translate digital designs into physical models, providing tangible representations of the intended grafts. The rapid prototyping process facilitates iterative design modifications, enabling surgeons to fine-tune graft specifications based on the unique requirements of each patient. This agility in the design and production phases streamlines the surgical workflow, reducing the time between preoperative planning and the actual procedure. Rapid prototyping through 3D printing exemplifies a dynamic and responsive approach to cartilage reconstruction, aligning with the principles of precision medicine and contributing to more effective and patient-specific interventions in otologic surgery [41].

*Advances in 3D Printing Technology:* *Bioink Development*

Cell-laden bioinks: Developing bioinks containing living cells represents a cutting-edge advancement in 3D bioprinting for cartilage reconstruction. Bioinks are specialized materials that can encapsulate living cells, allowing for the creation of bioprinted constructs with enhanced potential for tissue integration and regeneration. In the context of cartilage reconstruction, cell-laden bioinks enable the precise deposition of living cells in a controlled manner, mimicking the natural cellular composition of cartilage tissue. This approach fosters the creation of bioprinted grafts that provide structural support and actively contribute to the regenerative process by promoting cell proliferation and differentiation. Using cell-laden bioinks signifies a transformative shift toward biofabrication techniques incorporating living elements, enhancing the biomimicry and regenerative potential of 3D-printed cartilage constructs [42].

Vascularization strategies: Incorporating vascularization strategies into 3D-printed constructs addresses a fundamental challenge in tissue engineering, providing sufficient blood supply to support graft viability. In the context of cartilage reconstruction, ensuring adequate vascularization is crucial for nourishing and surviving transplanted tissues. 3D-printed constructs can be designed with intricate vascular networks, mimicking the natural blood supply of native tissues. This vascularization approach goes beyond structural support, actively contributing to the long-term success of the graft by promoting nutrient exchange and waste removal. The integration of vascularization strategies exemplifies a forward-thinking approach in 3D bioprinting, advancing the field toward creating more complex and physiologically relevant tissues for cartilage reconstruction [43].

Case studies and clinical outcomes

Illustrative Cases Demonstrating Successful Cartilage Application

Palisade cartilage tympanoplasty has emerged as a valuable technique for addressing perforations of varying sizes, boasting excellent graft take rates and favorable postoperative hearing outcomes, as indicated by a comprehensive systematic review and meta-analysis [44]. Beyond its effectiveness in primary cases, this technique has proven beneficial in addressing recurrent perforations, adhesive otitis media, tympanic membrane retractions, and other mixed middle ear pathologies [44]. In myringoplasty, a retrospective study encompassing 161 cases highlighted the prevalence of the retroauricular approach and identified tragal or conqual cartilage as the frequently utilized graft material [45]. Notably, concerns regarding cartilage stiffness were dispelled, with the study reporting satisfactory functional outcomes [45].

Another avenue in ear surgery involves cartilage reinforcement tympanoplasty, which demonstrated its efficacy in utilizing cartilage as a graft material for the closure of tympanic membrane perforations or the restoration of ossicles, particularly in cases of advanced middle ear disease [46]. Exploring the innovative realm of cartilage conduction hearing, studies have investigated its clinical applications in hearing perception through direct vibro-acoustic stimulation at various anatomical sites [47]. The development of monaural and binaural behind-the-ear cartilage conduction hearing aids has shown promise in enhancing hearing function, presenting a noteworthy advancement in the field [47]. Collectively, these diverse case studies and clinical outcomes underscore the versatility and effectiveness of cartilage in ear surgery. This multifaceted approach ensures excellent graft take rates and postoperative hearing outcomes. It addresses a spectrum of perforations and middle ear pathologies, showcasing cartilage as a valuable asset in otological interventions.

Long-Term Follow-up Studies on Patients Undergoing Cartilage-Based Procedures

Microfracture treatment has proven to yield positive clinical results, with a study reporting sustained efficacy even during long-term follow-up [48]. The utilization of a hyaluronic acid-based scaffold with bone marrow aspirate concentrate (HA-BMAC) in a case series of 23 patients demonstrated favorable long-term clinical outcomes for repairing full-thickness cartilage injuries in the knee, with assessments conducted over an average period of eight years (range: six to 10 years) [49]. In the context of matrix-assisted cartilage implantation (MACI), a study with a follow-up ranging from five to 12 years revealed failure rates of 18.2% and 87.5% in complex and salvage cases, respectively [50]. Autologous chondrocyte transplantation (ACT) exhibited encouraging results, with a study reporting consistent indications of cartilage regeneration over a seven-year follow-up period [51]. Examining intra-articular sprifermin in patients with knee osteoarthritis, a five-year follow-up study demonstrated the sustained structural modification of articular cartilage with sprifermin compared to a placebo over a 3.5 to four-year post-treatment period [52]. These comprehensive long-term follow-up studies collectively underscore the potential of cartilage-based procedures in providing enduring benefits in structural improvements and symptomatic relief for patients with various forms of joint disease. Nevertheless, the importance of continued long-term follow-up studies cannot be overstated, ensuring a comprehensive understanding of these procedures' enduring effectiveness and safety [51].

## Conclusions

In conclusion, this comprehensive review underscores cartilage's pivotal role in otologic surgery, serving as a versatile and resilient biological material with unique properties that contribute significantly to both cosmetic and functional outcomes. From reconstructing the external ear to reinforcing the tympanic membrane and restoring the ossicular chain, cartilage emerges as a fundamental component in addressing diverse structural deficiencies within the ear. Its biocompatibility, flexibility, and regenerative potential make it an indispensable resource for otologic surgeons seeking optimal solutions for their patients. Looking ahead, the review has highlighted groundbreaking advancements and emerging trends, particularly in tissue engineering, biodegradable scaffolds, and 3D printing technology. These innovations hold great promise for the future, offering more targeted and effective interventions that may redefine the landscape of otologic surgery. The convergence of these technologies, coupled with ongoing research and collaboration, is poised to elevate the standard of care, minimize complications, and ultimately enhance the quality of life for individuals undergoing cartilage-based otologic procedures.
